# Supplementary material for: Transmetatarsal amputations in patients with diabetes mellitus: A contemporary analysis from an academic tertiary referral centre in a developing community
Source: PLoS One. 2022 Nov 3;17(11):e0277117. doi: 10.1371/journal.pone.0277117 (PMC9632785; doi:10.1371/journal.pone.0277117)
Supplement: Attachment — Submitted filename: Editor Revision - TMA paper.docx [file pone.0277117.s002.docx]

Editor review

The paper is very interesting and well written and gives some evidence on the risk factors connected to complicated TMA and progression to major LEA. Tables are very explicative. The discussion would benefit from adding a few sentences regarding improvement of microcirculation after tarsal tunnel release in diabetic patients, and possible forefoot microvascular reconstruction in motivated patients. The bibliography can be further implemented. The paper can be accepted after minor revision.

Page 4, line 9

Please add sequent citation form the same Journal

[Coralie Amadou](https://pubmed.ncbi.nlm.nih.gov/?term=Amadou+C&cauthor_id=33253241) [^1^](https://pubmed.ncbi.nlm.nih.gov/33253241/#affiliation-1) [^2^](https://pubmed.ncbi.nlm.nih.gov/33253241/#affiliation-2), [Pierre Denis](https://pubmed.ncbi.nlm.nih.gov/?term=Denis+P&cauthor_id=33253241) [^3^](https://pubmed.ncbi.nlm.nih.gov/33253241/#affiliation-3), [Kristel Cosker](https://pubmed.ncbi.nlm.nih.gov/?term=Cosker+K&cauthor_id=33253241) [^3^](https://pubmed.ncbi.nlm.nih.gov/33253241/#affiliation-3), [Anne Fagot-Campagna](https://pubmed.ncbi.nlm.nih.gov/?term=Fagot-Campagna+A&cauthor_id=33253241) [^3^](https://pubmed.ncbi.nlm.nih.gov/33253241/#affiliation-3) Less amputations for diabetic foot ulcer from 2008 to 2014, hospital management improved but substantial progress is still possible: A French nationwide study- PLoS One. 2020 Nov 30;15(11):e0242524.

 doi: 10.1371/journal.pone.0242524. eCollection 2020. PMID: 33253241, PMCID: [PMC7703996](http://www.ncbi.nlm.nih.gov/pmc/articles/pmc7703996/), DOI: [10.1371/journal.pone.0242524](https://doi.org/10.1371/journal.pone.0242524)

Page 6, line 13

Please add following citation

[Low-vacuum negative pressure wound therapy protocol for complex wounds with exposed vessels.](https://pubmed.ncbi.nlm.nih.gov/35077217/) Paolini G, Sorotos M, Firmani G, Gravili G, Ceci D, Santanelli di Pompeo F. J Wound Care. 2022 Jan 2;31(1):78-85. doi: 10.12968/jowc.2022.31.1.78.PMID: 35077217

Page 12 line 14,

Please Add Citation and briefly discuss report about improvement of microcirculation after tarsal tunnel release in diabetic patients

[Evaluation of peripheral microcirculation improvement of foot after tarsal tunnel release in diabetic patients by transcutaneous oximetry.](https://pubmed.ncbi.nlm.nih.gov/25641727/)

Trignano E, Fallico N, Chen HC, Faenza M, Bolognini A, Armenti A, Santanelli Di Pompeo F, Rubino C, Campus GV.Microsurgery. 2016 Jan;36(1):37-41. doi: 10.1002/micr.22378. Epub 2015 Jan 13.PMID: 25641727

Page 12 line 14,

Please add Citation and briefly discuss report about possible forefoot microvascular reconstruction also in diabetic patients

[Microvascular reconstruction of complex foot defects, a new anatomo-functional classification.](https://pubmed.ncbi.nlm.nih.gov/26004168/)

Santanelli di Pompeo F, Pugliese P, Sorotos M, Rubino C, Paolini G. Injury. 2015 Aug;46(8):1656-63. doi: 10.1016/j.injury.2015.05.002. Epub 2015 May 12.PMID: 26004168
